# Sedation and sleep safety of pimavanserin for Parkinsons’s disease psychosis: Review and exploratory analysis of clinical study data

**DOI:** 10.1016/j.prdoa.2025.100342

**Published:** 2025-05-12

**Authors:** Ana Berrio, Lambros Chrones, Victor Abler, Robert A. Hauser

**Affiliations:** a Acadia Pharmaceuticals Inc., San Diego, CA, USA; b Parkinson’s Disease & Movement Disorders Center, University of South Florida, Tampa, FL, USA

**Keywords:** Parkinson’s disease psychosis, PDP, Hallucinations, Delusions, Sleep, Sedation, Somnolence, Orthostatic hypotension, Dizziness, Atypical antipsychotics

## Abstract

Patients with Parkinson’s disease almost uniformly experience sleep disorders. Sleep disorders have been linked to the severity of cognitive impairment, which is also a comorbidity with Parkinson’s disease psychosis (PDP). Pimavanserin, a highly selective inverse agonist of the serotonin 2A receptor, is approved in the United States for the treatment of hallucinations and delusions in patients with PDP. Other atypical antipsychotics, such as quetiapine and clozapine, are frequently used off-label to treat PDP and have an increased risk of sedation and other sleep-related adverse events (eg, drowsiness, sedation). By contrast, a high degree of specificity for the serotonin 2A receptor may prevent pimavanserin from causing some of the off-target adverse events associated with other atypical antipsychotics. In this review and exploratory analysis, we summarize pimavanserin sleep- and sedation-related safety and tolerability data from past clinical trials, including 6 randomized controlled trials and 2 open-label extensions representing various pimavanserin doses (1 to 51 mg) in healthy volunteers, patients with PDP, and older patients with neurodegenerative disease. The results from our exploratory analyses suggest that pimavanserin may be associated with negligible levels of sedation and other sleep‑related adverse events and may provide improvements in nighttime sleep compared with placebo. Future head-to-head clinical trials will be needed for direct comparisons between pimavanserin and other atypical antipsychotics, such as quetiapine or clozapine.

## Highlights

• This review summarizes pimavanserin sleep-related safety data from clinical trials.
• Pimavanserin may be associated with low levels of sleep‑related adverse events.
• Pimavanserin may also be associated with an improvement in nighttime sleep.
• Head-to-head trials are needed to compare pimavanserin with other antipsychotics.

## 1 Introduction

Parkinson’s disease is a common progressive neurodegenerative disease (NDD) characterized by motor symptoms of bradykinesia, rigidity, resting tremor, and disturbances of balance and posture [1]. Neuropsychiatric sequelae such as Parkinson’s disease psychosis (PDP), as well as dementia and sleep disturbances [1,2], also manifest at a cumulative prevalence of approximately 60 % [3]. Sleep disorders include excessive daytime sleepiness, insomnia, sleep fragmentation, nightmares, sleep apnea, and rapid eye movement (REM) sleep behavior disorder (RBD) [[4], [5], [6]] and are a major cause of disability in Parkinson’s disease, affecting 60 %‑98 % of patients [7].

Sleep disorders in patients with Parkinson’s disease are directly associated with the severity of cognitive impairment [[8], [9], [10], [11], [12]]. Multifactorial origins of sleep disturbances in this population may involve the direct or indirect effects of neuropathologic loss of dopaminergic neurons, deposition of α-synuclein, or side effects of Parkinson’s disease medications on other important neurotransmitter systems affecting sleep-wake regulation, sleep apnea, or RBD (reviewed in Samizadeh et al [13]). RBD is a strong risk factor and prodromal marker for developing Parkinson’s disease [14], and RBD or other sleep disturbances may increase the risk of hallucinations as patients develop PDP [15].

Pimavanserin, a serotonin 5‑HT_2A_ receptor inverse agonist/antagonist (with lesser affinity at 5-HT_2C_ receptors), is currently the only FDA‑approved treatment for hallucinations and delusions associated with PDP [[16], [17], [18]]. Other atypical antipsychotics are often used off-label to treat PDP and may have the risk of undesirable adverse effects (AEs) such as worsening parkinsonism [[19], [20], [21], [22], [23]]. The FDA has issued a class-wide boxed label warning for increased mortality risk with all atypical antipsychotics, including pimavanserin, in elderly patients with dementia [22,24]. Patients with PDP are often older, frail adults experiencing high rates of daytime somnolence and sleep attacks [25]. These patients also tend to have high rates of polypharmacy, which increases the potential for drug-drug interactions and AEs from other medications [26].

Sedation is a prominent AE of atypical antipsychotics, including quetiapine and clozapine [20,27,28]. Other AEs frequently reported for quetiapine and clozapine include somnolence, drowsiness, dizziness, and orthostatic hypotension [[28], [29], [30]]. Drowsiness and somnolence may be manifestations of the same phenomenon (sleepiness), with somnolence being a more severe state of near-sleep potentially interfering with activities of daily living. The terms drowsiness and somnolence are sometimes used interchangeably without attention to the nuanced differences in meaning, though studies usually report one or the other. They may have many triggers including medication, psychiatric disorders, or sleep deprivation. Sedation refers to a state of calm typically only associated with medication. Sedation and hypotension may be related to the inhibitory effects of these drugs on histaminergic and adrenergic receptors, respectively [28,31,32]. Such limiting AEs further increase the risk of falls and fractures in the population of patients with PDP [26,33]. Unlike other atypical antipsychotics, pimavanserin does not have activity at dopaminergic, histaminergic, muscarinic, and adrenergic receptors [22,34]. Due to this high degree of specificity, pimavanserin is not associated with many of these off-target AEs [33].

Evidence has shown that patients with PDP are sensitive to sleep- and sedation-related AEs associated with most atypical antipsychotics [33,35,36]. Given these findings, we conducted this analysis to summarize sleep- and sedation-related safety and tolerability data from past trials of pimavanserin, which includes both previously published and unpublished results.

## 2 Methods

### 2.1 Study selection

Pimavanserin safety data were gathered from 8 previously completed placebo-controlled clinical studies with various exploratory outcome measures relevant to sleep and sedation (Table1). These include the following: ACP-103–011 [37]; ACP-103–006/CONCEPT 2 (NCT00087542) [31] and its corresponding open-label extension (OLE) ACP-103–010 (NCT01518309); ACP-103–012 (NCT00477672) [38]; ACP-103–014 (NCT00658567); ACP-103–020 (NCT01174004) [39], which was the FDA registration trial for pimavanserin; ACP-103–015 (NCT00550238), the OLE for studies 012, 014, and 020; and ACP-103–046 (NCT03575052) [18]. For studies with previously published study designs or results, pimavanserin dosages are sometimes presented as pimavanserin tartrate and sometimes as equivalent pimavanserin free base. Doses of pimavanserin tartrate correspond to those of pimavanserin free base as follows: 10 mg pimavanserin tartrate is equivalent to 8.5 mg pimavanserin free base; 20 mg pimavanserin tartrate is 17 mg free base; and 40 mg pimavanserin tartrate is 34 mg free base [16,22,38]. Unless otherwise specified, pimavanserin doses mentioned herein are reported as pimavanserin free base, in alignment with the label.

**Table 1:** Pimavanserin studies included for safety and sleep analysis.

**Study**	**Description**	**Sedation/sleep exploratory end points**
Healthy adults ACP-103–011 [37]	Phase 1, 2-week, double-blind, randomized study of older (mean age > 51 years), healthy adults (N = 45) randomized to pimavanserin (1, 2.5, 5, or 20 mg^a^ in the morning) or placebo	• Nighttime sleep quality (SWS measured by polysomnography)
Patients with PDP ACP-103–006/CONCEPT 2 (NCT00087542) [31]	Phase 2, 8-week (4 weeks of double-blind treatment), dose-escalation study of patients with PDP (N = 60) randomized to pimavanserin (17 mg with a possible increase to 34 or 51 mg) or placebo with an OLE (ACP-103–010)	• Lightheadedness/ dizziness and nighttime sleep quality (Symptom Questionnaire) • Situational sleepiness (ESS)
Patients with PDP, ≥6-week treatment duration^a^ ACP-103–012 (NCT00477672) [38] ACP-103–014 (NCT00658567) CP-103–020 (NCT01174004) [39]	Phase 2b/3, 6-week, international, randomized study of patients with PDP (N = 298) randomized to pimavanserin (8.5 mg or 34 mg)^a^ or placebo	• Nighttime sleep quality and daytime sleepiness (SCOPA-Sleep) • TEAEs
	Phase 3, 6-week, randomized, double-blind study of patients with PDP (N = 199) randomized to pimavanserin (34 mg)^a^ or placebo with an OLE (ACP-103–015)	• Nighttime sleep quality and daytime sleepiness (SCOPA-Sleep) • TEAEs
Patients with PDP, OLE studies^b^ ACP-103–010 (NCT01518309) and ACP-103–015 (NCT00550238)	OLE of CONCEPT 2 (ACP-103–006) (N = 39)	• TEAEs
	OLE of the phase 3, 6-week study (ACP-103–020) (N = 459)	• TEAEs
Older participants with NDD ACP-103–046 (NCT03575052) [18]	Phase 3b, 8-week treatment (study duration of up to 16 weeks), multicenter, randomized, double-blind, placebo-controlled, two-arm, parallel-group study of patients with neuropsychiatric symptoms related to NDD^c^ (N = 784) randomized to pimavanserin (34 mg) or placebo	• Sleep disturbance (SDI)

ESS, Epworth Sleepiness Scale; ISS, integrated safety summary; NDD, neurodegenerative disease; OLE, open-label extension; PDP, Parkinson’s disease psychosis; SCOPA-Sleep, Scales for Outcomes in Parkinson’s Disease – Sleep; SDI, Sleep Disorders Inventory; SWS, slow-wave sleep; TEAEs, treatment-emergent adverse events.

a 20 mg pimavanserin tartrate is equivalent to 17 mg pimavanserin free base.

b An integrated summary of the safety study was conducted, which consisted of the ≥ 6-week treatment duration studies (ACP-103–012, ACP-103–014, and ACP-103–020) and the OLE studies (ACP-103–010 and ACP-103–015).

c NDDs assessed included Alzheimer’s disease, vascular dementia, Parkinson’s disease (with or without dementia), frontotemporal dementia, and dementia with Lewy bodies.

All pimavanserin clinical studies were performed in accordance with the ethical principles of the Declaration of Helsinki, Good Clinical Practice guidelines, and other applicable and local regulatory requirements. Study protocols were approved by Independent Ethics Committees or Institutional Review Boards. Patients provided written informed consent prior to participating in each study.

Articles describing sleep-related effects of quetiapine and clozapine were added to this review to provide context for pimavanserin data. Articles were sourced using PubMed searches, with an emphasis on systematic literature reviews and clinical study publications. Preference was placed on articles that contained data similar to existing pimavanserin data.

### 2.2 Study designs

#### 2.2.1 Pimavanserin in healthy adults

The study design and results for ACP-103–011 have been previously published [37]. Briefly, this was a phase 1, single-center, placebo-controlled, double-blind study to assess the effect of pimavanserin tartrate (1, 2.5, 5, or 20 mg) on slow-wave sleep in older (mean age > 51 years), healthy adults (N = 45). Nighttime sleep quality was assessed as slow-wave sleep duration (primary end point) measured by polysomnography at baseline and days 1 and 13 [37]. Other sleep-related end points included sleep period time, total sleep time, sleep onset latency, number of stage shifts, total time awake, early morning wake, microarousal index, number of awakenings after sleep onset, and duration of awakenings [37].

#### 2.2.2 Pimavanserin in PDP

ACP-103–006 (CONCEPT 2; NCT00087542) was a phase 2, placebo-controlled, double-blind, dose-escalation study of patients with PDP (N = 60) randomized to pimavanserin tartrate at 20 mg with possible increase to 40 or 60 mg or placebo [31]. The trial consisted of a 4-week double-blind treatment period and a 4-week follow-up period. The full study design, plus the primary assessment of parkinsonism symptoms and select secondary measures for the per-protocol population of ACP-103–006, have been described previously [31].

The safety population included all patients who received ≥ 1 dose of the study drug (N = 60; pimavanserin, n = 29; placebo, n = 31), and patients were classified according to the treatment they received [31]. The intent-to-treat (ITT) population included all randomized participants who met entry criteria, received ≥ 1 dose of the study drug, and had ≥ 1 efficacy outcome measure.

Daytime sleepiness as an exploratory end point in this study was measured by the Epworth Sleepiness Scale (ESS) at baseline and week 4, and ESS change from baseline to week 4 for the per-protocol population was previously reported [31]. Here, the total ESS scores at week 4 and change from baseline to week 4 are shown from all 3 efficacy populations (ITT, modified ITT, and per-protocol populations) of study ACP-103–006. Additional sleep-related safety measures analyzed herein are from the safety population and include the symptom questionnaire for lightheadedness/dizziness and sleep quality at weeks 4 and 8, as well as select treatment-emergent adverse events (TEAEs) in ≥ 2 % of participants.

The OLE for this study, ACP-103–010 (NCT01518309), enrolled 39 patients who had previously completed a blinded study with pimavanserin and were considered by their treating physician potentially to benefit from continued treatment.

ACP-103–012 (NCT00477672) [38], ACP-103–014 (NCT00658567), and ACP-103–020 (NCT01174004) [39] were phase 3, 6-week, randomized, placebo-controlled, double-blind studies evaluating pimavanserin to reduce the frequency and severity of hallucinations and delusions in patients with PDP. Study ACP-103–012 enrolled 298 who were randomized to receive 8.5 or 34 mg pimavanserin or placebo. Study design and sleep-related outcome measures for this study have been previously reported [38]. Another international study, ACP-103–014, evaluated 8.5 or 17 mg of pimavanserin vs placebo in patients with PDP. After enrolling 123 patients, study ACP-103–014 was stopped early following the results of study ACP-103–012, which did not support efficacy at 8.7 mg of pimavanserin in this population. Study ACP-103–020, the pivotal trial supporting FDA approval of pimavanserin, evaluated patients with PDP (N = 199) randomized to 34 mg pimavanserin or placebo, as previously described [39]. Exploratory end points for these studies included nighttime sleep quality and daytime sleepiness, as measured by Scales for Outcomes in Parkinson’s Disease–Sleep (SCOPA-Sleep) at baseline and weeks 1, 2, 4, and 6.

Pooled analyses of the 34-mg pimavanserin and placebo treatment arms of studies ACP-103–012 and ACP-103–020 were previously performed to evaluate the effect of pimavanserin on sleep outcomes [38]. As part of these analyses, patients with significant baseline sleepiness at nighttime (SCOPA-NS ≥ 7) and daytime (SCOPA-DS ≥ 5) were assessed for treatment effects [38].

Eligible patients in studies ACP-103–012 (NCT00477672), ACP-103–014 (NCT00658567), and ACP-103–020 (NCT01174004) (N = 459) participated in an OLE (ACP-103–015; NCT00550238), for which the study design has been described [40,41] and is summarized in Table1. In addition, an integrated summary of the safety study was conducted, which consisted of the ≥ 6-week treatment duration studies (ACP-103–012, ACP-103–014, and ACP-103–020) and the OLE studies (ACP-103–010 and ACP-103–015).

#### 2.2.3 Pimavanserin in NDD

Study ACP-103–046 (NCT03575052) was a phase 3b, 8-week treatment (study duration of up to 16 weeks), multicenter, randomized, double-blind, placebo-controlled study of older patients (N = 784) with neuropsychiatric symptoms related to NDD who were randomized to receive 34 mg pimavanserin or placebo [18]. Study design and results have been previously published [18]. Briefly, NDDs assessed in this study included Alzheimer’s disease, vascular dementia, Parkinson’s disease (with or without dementia), frontotemporal dementia, and dementia with Lewy bodies. Sleep disturbances as an exploratory end point were assessed using the Sleep Disorders Inventory (SDI) at baseline and weeks 4 and 8 [18]. These data were analyzed using the mixed-effects model repeated measures (MMRM) and are presented as least-square means (LSM) ± standard error of change in baseline at weeks 4 and 8.

### 2.3 Statistics

Statistical information from defined trial populations is presented where available and specific for each trial protocol and prespecified analysis population. Pooled data from the post hoc analyses of the integrated safety study were not powered for statistical comparisons.

## 3 Results

### 3.1 Pimavanserin in healthy adults

In a study of 45 healthy volunteers (ACP‑103‑011), pimavanserin tartrate (5 mg and 20 mg) was associated with significant (*P* < 0.001) increases in slow‑wave sleep duration compared with placebo (Table2) [37]. This effect occurred after only 1 treatment (day 1) and was sustained for 13 days of pimavanserin administration, with a similar treatment effect across the 2 assessment days [37]. Slow-wave sleep duration was positively correlated with plasma levels of pimavanserin (r, 0.51; 95 % CI, 0.22–0.72; r^2^, 0.26, *P* = 0.002), suggesting a dose-dependent effect of pimavanserin shifting to deeper nighttime sleep [37].

**Table 2:** Effects of pimavanserin versus placebo on duration (minutes) of slow-wave sleep^a^ in healthy adults (ACP-103–011).

**Time following treatment**	**Pimavanserin**^b^				**Placebo** **(n = 9)**
	**1 mg** **(n = 9)**	**2.5 mg** **(n = 9)**	**5 mg** **(n = 9)**	**20 mg**^b^ **(n = 9)**	
**Baseline**	63.39 ± 26.12	54.00 ± 30.88	57.61 ± 33.06	83.94 ± 21.44	67.39 ± 32.06
**Day 1**	69.78 ± 32.14	75.83 ± 50.91	94.89 ± 39.84	129.94 ± 36.29	56.22 ± 19.12
DLS mean^c^	17.3	32.4	48.1	57.9	
*P* value^d^	0.178	**0.013**	**<0.001**	**<0.001**	
**Day 13**	68.78 ± 31.01	73.61 ± 50.82	99.56 ± 49.64	122.94 ± 44.69	64.28 ± 13.96
DLS mean^c^	8.3	22.2	44.7	42.8	
*P* value^d^	0.519	0.088	**<0.001**	**0.001**	
**Overall treatment effect**					
DLS mean^c^	12.8	27.3	46.4	50.4	
*P* value^e^	0.160	**0.004**	**<0.001**	**<0.001**	

Mean ± standard deviation of slow-wave sleep duration (minutes) for each time point is shown. The overall ANCOVA for treatment was *P* < 0.001. Abbreviations: ANCOVA, analysis of variance; DLS, difference between least-squares.

a Slow-wave sleep was measured via polysomnography.

b Dosages represent pimavanserin tartrate; 20 mg pimavanserin tartrate is equivalent to 17 mg pimavanserin free base.

c The DLS mean represents the difference between least-squares means for each pimavanserin and placebo dose (pimavanserin-placebo).

d The *P* value for the treatment effect from the ANCOVA model at the time point.

e The *P* value for the overall treatment effect (averaged over study days 1 and 13).

The study also reported significant improvements in secondary end points of parameters related to sleep continuity, sleep architecture, and sleep profile as analyzed by multivariate analysis of variance. Specifically, pimavanserin significantly decreased the overall number of awakenings after sleep onset (*P* < 0.05; sleep continuity), increased non-REM sleep duration (*P* < 0.05) and proportion of a whole night’s sleep (*P* < 0.01; sleep architecture), and increased the duration of slow-wave sleep in the first (*P* < 0.01) third of the night and the second (*P* < 0.001) third of the night, but not the last third of the night [37]. Pimavanserin did not alter total sleep time, indicating that, while REM sleep was not affected, the increase in slow-wave sleep came at the expense of other non-REM stages. This is evident in the highest 2 doses, where the duration of stage 2 was significantly reduced [37]. Pimavanserin did not affect sleep onset latency in this study, a finding consistent with a lack of soporific effect.

Sleep-related TEAEs reported in this phase 1 study included 2 volunteers in the 5-mg pimavanserin group noting “deeper sleep” and 1 in the 1-mg pimavanserin group noting “increased dreams”; no participants in the placebo group reported changes in sleep-related events [37].

### 3.2 Pimavanserin in patients with PDP

#### 3.2.1 ACP-103–006/CONCEPT 2

In the per-protocol population (N = 52) of the phase 2 CONCEPT 2 trial (ACP‑103‑006), pimavanserin (≥17 mg) demonstrated slight but not significant improvements in daytime sleepiness by difference in least-squares means of ESS scores (−0.9; 95 % CI: −3.3, 1.5; *P* = 0.46) [31]. Our analysis of ESS scores in the ITT population (groups reported as assigned) and mITT population (groups reported as received) also showed similarities between the pimavanserin (n = 28) and placebo (n = 31) groups (Table3). Dizziness, nighttime sleep quality, and excessive daytime sleepiness assessed by symptom questionnaire in the safety population were also similar between the pimavanserin (n = 29) and placebo (n = 31) groups (Table3).

**Table 3:** Dizziness, nighttime sleep quality, and sleepiness in patients with PDP treated with pimavanserin or placebo in study ACP-103–006.

**Events and severity, n (%)**	**Pimavanserin** **(n = 29)**		**Placebo** **(n = 31)**	
	**Week 4**	**Week 8**	**Week 4**	**Week 8**
**Symptom Questionnaire (safety population)**				
Lightheadedness/dizziness				
None	14 (48.3)	6 (20.7)	9 (29.0)	10 (32.3)
Mild	9 (31.0)	12 (41.4)	13 (41.9)	10 (32.3)
Moderate	2 (6.9)	2 (6.9)	7 (22.6)	4 (12.9)
Severe	0	0	0	0
Poor sleeping at night				
None	7 (24.1)	7 (24.1)	13 (41.9)	12 (38.7)
Mild	5 (17.2)	5 (17.2)	6 (19.4)	6 (19.4)
Moderate	11 (37.9)	6 (20.7)	5 (16.1)	4 (12.9)
Severe	2 (6.9)	2 (6.9)	5 (16.1)	2 (6.5)
Excessive daytime sleepiness				
None	5 (17.2)	4 (13.8)	7 (22.6)	4 (12.9)
Mild	6 (20.7)	5 (17.2)	11 (35.5)	10 (32.3)
Moderate	13 (44.8)	9 (31.0)	9 (29.0)	8 (25.8)
Severe	1 (3.4)	2 (6.9)	2 (6.5)	2 (6.5)
**ESS (ITT population)**^a^	**(n = 28)**		**(n = 31)**	
Total score, mean (SE)	13.1 (0.87)		11.5 (0.99)	
Change from baseline to week 4	−0.5 (0.79)		−0.3 (0.70)	
**ESS (modified ITT population)**^b^	**(n = 28)**		**(n = 31)**	
Total score, mean (SE)	12.9 (0.92)		11.7 (0.96)	
Change from baseline to week 4	−0.9 (0.74)		0.0 (0.73)	
**ESS (per-protocol population)**^c^	**(n = 24)**		**(n = 28)**	
Total score, mean (SE)	13.2 (0.97)		11.7 (1.01)	
Change from baseline to week 4	−0.9 (0.86)		−0.3 (0.78)	

ESS data were analyzed via LOCF with ANCOVA model performed to compare the change from baseline between the 2 groups. Abbreviations: ESS, Epworth Sleepiness Scale; ITT, intent to treat; LOCF, last observation carried forward; SE, standard error.

a ITT population was classified according to the treatments originally assigned and was defined as all randomized participants who had met entry criteria and had taken at least 1 dose of trial medication and at least 1 postbaseline efficacy outcome measure.

b Modified ITT population included participants from the ITT population who were classified according to treatment received. This included 1 patient who had been assigned to the pimavanserin group but received a placebo and another who had been assigned to the placebo group but received pimavanserin.

c Per-protocol population included participants from the ITT population who had met entry criteria and recorded no major deviations from protocol, including completion of the week 4 visit with at least 70% overall treatment compliance. This population was classified according to the treatment received.

#### 3.2.2 ACP-103–012 and ACP-103–020

**Fig. 1:**
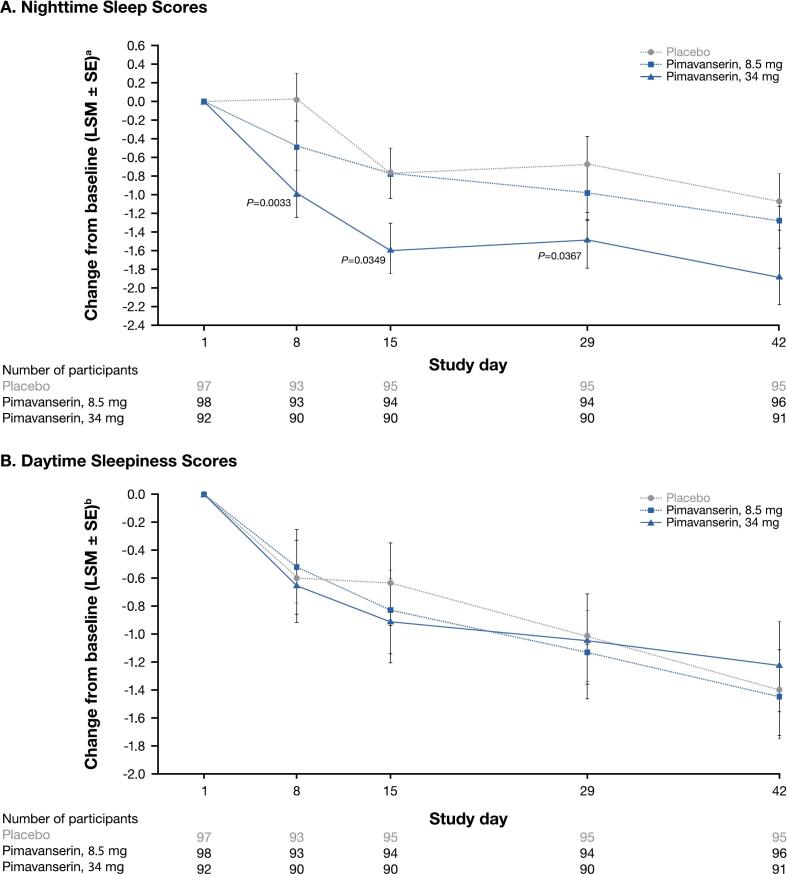
Change from baseline to week 6 (day 42) for pimavanserin (8.5 and 34 mg) and placebo groups for the SCOPA-Sleep (A) nighttime^1^ sleep and (B) daytime sleepiness^2^ scores (ACP-103–012; ITT population). ITT, intent to treat; LSM, least-squares mean; SCOPA-Sleep, Scales for Outcomes in Parkinson’s Disease – Sleep; SE, standard error. Missing data were corrected using the last observation carried forward method. ^1^SCOPA-Sleep nighttime sleep score range is 0 to 15, where a negative change in score indicates improvement in nighttime sleep. ^2^SCOPA-Sleep daytime sleep score range is 0 to 18, where a negative change in score indicates improvement in sleepiness.

**Fig. 2:**
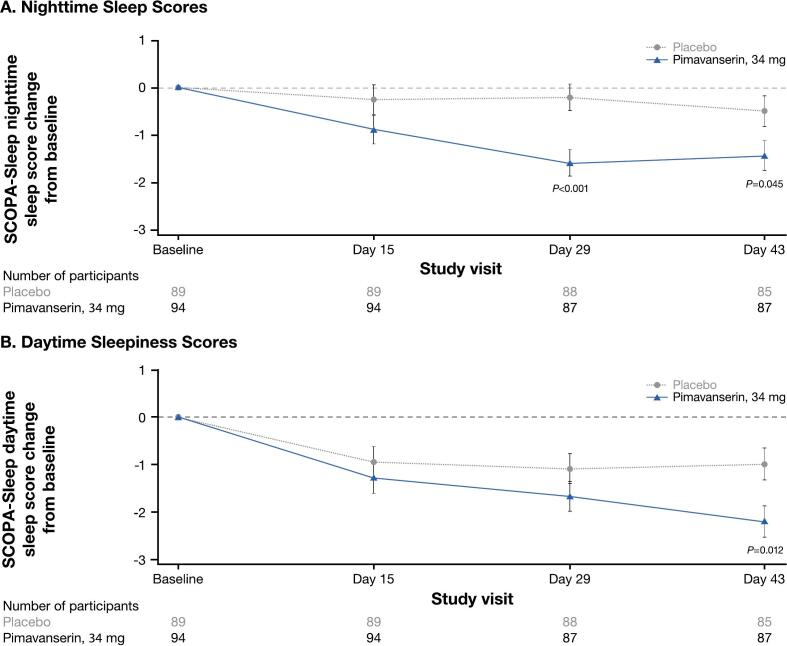
Change from baseline to week 6 for pimavanserin (34 mg) and placebo groups for the SCOPA-Sleep (A) nighttime^1^ and (B) daytime sleepiness^2^ scores (ACP-103–020; ITT population). ITT, intent to treat; LSM, least-squares mean; SCOPA-Sleep, Scales for Outcomes in Parkinson’s Disease – Sleep; SE, standard error. ^1^SCOPA-Sleep nighttime sleep score range is 0 to 15, where a negative change in score indicates improvement in nighttime sleep. ^2^SCOPA-Sleep daytime sleepiness score range is 0 to 18, where a negative change in score indicates improvement in sleepiness.

As previously reported for ACP‑103‑012 [38], significant improvements with pimavanserin vs placebo were observed in nighttime sleep (Fig. 1**A**) but not daytime sleepiness (Fig. 1**B**) scores. In the pivotal trial ACP‑103‑020, patients with PDP taking pimavanserin demonstrated significant improvements over those in the placebo group in both nighttime (Fig. 2**A**) and daytime (Fig. 2**B**) SCOPA-Sleep scores [39]. The pooled analysis of ACP-103–012 and ACP-103–020 (placebo, n = 187; 34-mg pimavanserin, n = 186) was consistent with findings from the individual studies, with significant improvement over placebo in scores for nighttime sleep, but not daytime sleepiness, at weeks 2, 4, and 6 (LSM reduction at week 6: −1.8 for pimavanserin; −1.0 for placebo; *P* < 0.005) [38].

##### 3.2.2.1 Integrated safety summary

We conducted an integrated safety analysis with pooled data from past pimavanserin studies with treatment durations that were ≥ 6 weeks (ACP-103–012, ACP-103–014, and ACP-103–020) and OLE studies (ACP-103–010 and ACP-103–015). Of 383 patients receiving at least 1 dose of pimavanserin (8.5, 17, or 34 mg) across 3 randomized controlled trials, somnolence was reported in 11 (2.9 %), insomnia in 10 (2.6 %), orthostatic hypotension in 6 (1.6 %), and dizziness in 17 (4.4 %). Comparable rates were observed in 184 of these patients who received 34  mg pimavanserin in the 2 OLE studies. In the pooled placebo arm (n = 231), somnolence was reported in 6 (2.6 %), insomnia in 7 (3.0 %), orthostatic hypotension in 12 (5.2 %), and dizziness in 10 (4.3 %) individuals (Table4). Thus, rates of sleep-related TEAEs were similar between the pimavanserin and placebo arms in this integrated safety summary. Discontinuation of pimavanserin due to any TEAE occurred in 28 of 383 patients (7.3 %), of which the only sleep-related TEAEs were 1 case each (0.3 %) of hypersomnia and syncope.

**Table 4:** Sleep-related TEAEs occurring in ≥ 2 % participants (all pimavanserin or placebo groups) from studies with ≥ 6-week treatment duration and partial data from OLE studies.^a^

**Events, n (%)**	**All pimavanserin dosages** **(8.5, 17, and 34 mg)** **(n = 383)**	**Pimavanserin** **34 mg OLE**^b^ **(n = 184)**	**Placebo** **(n = 231)**
Somnolence	11 (2.9)	4 (2.2)	6 (2.6)
Insomnia	10 (2.6)	4 (2.2)	7 (3.0)
Orthostatic hypotension	6 (1.6)	4 (2.2)	12 (5.2)
Dizziness	17 (4.4)	3 (1.6)	10 (4.3)

OLE, open-label extension; TEAE, treatment-emergent adverse events.

a An integrated summary of the safety study was conducted, which consisted of the ≥ 6-week treatment duration studies (ACP-103–012, ACP-103–014, and ACP-103–020) and the OLE studies (ACP-103–010 and ACP-103–015).

b Includes adverse events up to day 72 for participants in OLE study 015 (ACP-103–015) that were in the placebo-treatment group in the ≥ 6-week treatment duration studies (ACP-103–012/ACP-103–014 and ACP-103–020).

### 3.3 Pimavanserin in patients with NDD

**Fig. 3:**
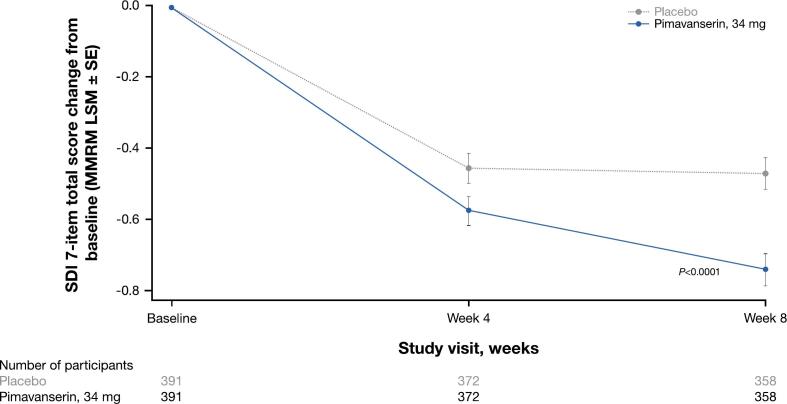
Change from baseline to weeks 4 and 8 for pimavanserin and placebo groups for the SDI total score (ACP-103–046) [18]. *P* < 0.0001, MMRM LSM difference (SE) at week 8: −0.3 (0.06); LSM from MMRM with fixed categorical effects of the region (ie, North America, Europe, or rest of world), planned treatment, visit, treatment-by-visit interaction, and fixed continuous covariates of baseline SDI score and baseline SDI score-by-visit interaction. LSM, least-squares means; MMRM, mixed-effects model repeated measures; SDI, Sleep Disorders Inventory; SE, standard error.

Study ACP-103–046 compared pimavanserin to placebo in patients with neuropsychiatric symptoms related to NDD (n = 392 patients per treatment group). Pimavanserin was associated with significant improvements in total SDI score change from baseline compared with placebo at week 8 (MMRM LSM, −0.3; SE, 0.06; *P* < 0.0001; Fig. 3) [18]. Of TEAEs occurring in ≥ 1 % of patients, dizziness was reported in 6 (1.5 %) patients in the pimavanserin group and 3 (0.8 %) in the placebo group [18].

### 3.4 Other atypical antipsychotics

Typical and atypical antipsychotics frequently cause sedation, which is more pronounced in older populations [28]. In addition to serotonin receptor (5-HT2) antagonism, many atypical antipsychotics, including quetiapine and clozapine, albeit to a lesser extent, have additional inhibitory activity on dopaminergic type 2 (D2) and histaminergic receptors [42]. Sedation may be related to dosage and histaminergic receptor affinity [28,42]. Inhibition of dopaminergic D2 receptors in the dorsal striatum reduces the effects of dopamine agonists and L-DOPA on motor symptoms [31,43]. Thus, this pharmacologic activity at dopaminergic receptors can complicate treatment of Parkinson’s disease. Currently, guidelines, including American Geriatrics Society (AGS) Beers Criteria, recommend not prescribing atypical antipsychotics for PDP, except quetiapine, clozapine, and pimavanserin [10].

Quetiapine is FDA approved to treat schizophrenia and bipolar disorder, but not PDP. Several early open-label studies were promising for quetiapine in the treatment of PDP, but subsequent randomized controlled trials did not demonstrate efficacy [23]. Nevertheless, it is the most frequently used atypical antipsychotic in the treatment of PDP [44,45]. Compared to pimavanserin, more individuals taking quetiapine for PDP do so for secondary symptoms, such as agitation or insomnia [46]. Quetiapine, especially at lower doses, is generally considered more tolerable than other atypical antipsychotics, possibly due to lower dopamine receptor occupancy at clinical doses [47,48] and similar effects on motor function compared to placebo [49]. Nevertheless, it retains a high incidence of sedation and somnolence [10,29] and is commonly used off-label to treat insomnia in elderly patients [29]. A systematic review of quetiapine in 19 studies across indications reported drowsiness (35 %), somnolence (25 %–39 %), dizziness (15 %–27 %), and sedation (8 %–11 %) among the most common AEs, frequently at high enough severity to warrant discontinuation (2.2 %–13 % discontinuation for drowsiness, somnolence, or dizziness) [29]. Reporting of cardiovascular AEs varies between studies and is likely due to variation in quetiapine dose [29]. While doses are titrated from 12.5 mg to 50–150 mg when treating PDP, dose dependency of AEs such as orthostatic hypotension has not been established [49].

Clozapine is approved in the US for treatment-resistant schizophrenia and reducing suicidal behavior in patients with schizophrenia. It was the first atypical antipsychotic with demonstrated efficacy in improving hallucinations and delusions in PDP without worsening any motor-related symptoms and significantly improving resting tremor [[50], [51], [52]]. Clozapine demonstrated greater efficacy than quetiapine at controlling delusions (*P* = 0.011) but not hallucinations (*P* = 0.097) in PDP in a head-to-head comparator study [53]. Despite this efficacy and regulatory approvals for patients with PDP in several other countries, clozapine has not been approved by the FDA for patients with PDP in the US [54]. Its utility is limited, particularly due to the increased risk of agranulocytosis; providers in the US are required to comply with extensive safety regulations to manage this risk [45]. In addition, sedation is among the most common clozapine AEs, with an incidence of approximately 44 %, and is thought to be dosage dependent [30]. Although clozapine is prescribed at much higher doses for schizophrenia (300–800 mg/day) than for PDP (6.25–50 mg/day) [54], one early study in patients with PDP taking lower dosages of clozapine reported somnolence in up to 53 % of patients and orthostatic hypotension in up to 19 % [52]. Sedation is one of the AEs most frequently cited as a reason for discontinuation of clozapine across indications (15.9 % and 21.5 % in 2 different retrospective studies) [55,56].

## 4 Discussion

For all sleep parameters and doses investigated in the reported pimavanserin clinical trials or analyzed herein, pimavanserin improved or did not affect sleep-related measures compared with placebo. In healthy volunteers, pimavanserin significantly improved slow‑wave sleep duration and other sleep-related parameters [37]. In patients with PDP, significant improvements with pimavanserin were observed in nighttime [38,39] and daytime SCOPA-Sleep scores [39]. Further safety analyses conducted for this review confirmed that treatment with pimavanserin did not worsen other sleep-related parameters (eg, ESS scores, dizziness, nighttime sleep quality, or sleepiness) or TEAEs. In patients with neuropsychiatric symptoms related to NDD, treatment with pimavanserin vs placebo significantly improved SDI scores [18]. Together, these data and post hoc analyses suggest that pimavanserin improves sleep architecture without causing sedation or worsening other sleep-related AEs.

These findings are important given the burden of sleep disturbances in Parkinson’s disease and their direct association with cognitive impairment and hallucinations [[8], [9], [10], [11], [12]]. Of the other atypical antipsychotics used off-label to treat PDP, quetiapine is the most frequently prescribed [57], as others such as risperidone, olanzapine, and ziprasidone commonly cause intolerable exacerbation of parkinsonism [10,22,23]. Quetiapine has not sufficiently demonstrated efficacy in patients with PDP [49]. Clozapine is efficacious in PDP and is unlikely to exacerbate parkinsonism [10] but requires burdensome monitoring for agranulocytosis in the US, and it has other AEs that raise tolerability concerns. Sleep-related AEs including sedation and orthostatic hypotension, which have been observed with quetiapine and clozapine, remain a concern, particularly in the PDP population [20,[27], [28], [29], [30]]. While the sedative effects of quetiapine or clozapine are occasionally warranted (eg, easing caregiver burden for nonmobile older patients), they further increase the risk of falls and fractures in this population [25,33] and can significantly impact their health-related quality of life. Nonetheless, the effectiveness of quetiapine and clozapine as off-label treatments for insomnia may be a reflection of their sedating effects [58]. Hallucinations in PDP are more common at night [59]; a reduction in nighttime awakenings through sedation reduces the opportunities for such hallucinations.

Pimavanserin’s high degree of specificity for the serotonin 5‑HT_2A_ and 5‑HT_2C_ receptors may mitigate the sleep-related AEs associated with other antipsychotics [33]. Quetiapine and clozapine both retain inhibitor activity on histaminergic receptors linked to sedation and adrenergic receptors linked to hypotension [28,31,32]. In addition, quetiapine and clozapine display residual inhibitory activity at dopaminergic receptors, which can decrease the motor benefit from dopaminergic medications [42]. Thus, unlike quetiapine and clozapine, pimavanserin is effective at treating psychotic symptoms while not worsening sleep quality and daytime sleepiness.

Although no head-to-head clinical trials comparing pimavanserin directly with quetiapine or clozapine have yet been published to our knowledge, ongoing studies are expected to be completed within the next 2–5 years (NCT04373317 and NCT05590637). In addition, a late-breaking abstract at MDS 2024 reported preliminary findings from a phase 3 prospective study of pimavanserin vs quetiapine in patients with PDP conducted by Sun Pharma Laboratories (Mumbai, India). Pimavanserin reportedly demonstrated noninferiority to quetiapine in mean change in SAPS-PD score and significantly improved SCOPA scores vs quetiapine for nighttime sleep (day 14) and daytime wakefulness (day 28) in the study population [60].

The clinical meaningfulness of the changes in sleep scales associated with pimavanserin may be low. The minimum clinically important difference (MCID) in SDI total score is 3 points [61], which is larger than what was demonstrated in study ACP-103–046 [18]. The MCID for the SCOPA-Sleep scale is not established to the best of our knowledge.

This review and exploratory post hoc analysis have some limitations. The lack of head-to-head studies with pimavanserin vs quetiapine or clozapine limits comparative analyses between the drugs. Anticipated publication of such studies may inform future comparative analyses between these atypical antipsychotics. Literature searches for this narrative review were primarily conducted using the PubMed database and are not comprehensive. Results of new pimavanserin analyses reported herein should be interpreted with caution because the data were analyzed post hoc from various prior clinical studies of pimavanserin.

Taken together, sleep-related data from clinical trials in healthy adults, patients with PDP, or patients with NDD suggest that pimavanserin may be associated with low levels of sedation and other sleep‑related adverse events, as well as improvements in nighttime sleep and sleep architecture.

## Disclosures

AB, LC, and VA are employees of 10.13039/100009427Acadia Pharmaceuticals, Inc., the manufacturer of pimavanserin. 10.13039/501100004267RAH has received speaking fees from 10.13039/100020357Amneal Pharmaceuticals, 10.13039/100019850Cerevel, Kyowa Kirin, 10.13039/100014593Neurocrine Biosciences, and Supernus; consulting fees from AbbVie, 10.13039/100020357Amneal, Avanex, 10.13039/100005614Biogen, BlueRock, 10.13039/100019850Cerevel, Forsee Pharmaceuticals, Inhibikase, Intrance (10.13039/100014275PSG), 10.13039/100011096Jazz Pharmaceuticals, Kiefe RX, Kyowa Kirin, MDCE Suzhou, MedRythms, 10.13039/100030838Merz, Mitsubishi Tanabe, Neurocrine, Neuroderm, Pharma Two B, Regenxbio, 10.13039/100015516Revance, 10.13039/100020461Serina Therapeutics, Stoparkinson, Supernus, Tolmar, Tremor Research Group, Tris Pharma, Truebinding, UCB, Vivifi, and Zambon; serves on a scientific advisory board for Stoparkinson and Inhibikase; holds stock in 10.13039/100015516Revance Therapeutics; has stock options in Enterin, Inhibikase, and Axial Therapeutics; has received intellectual property interests from a PD diary through his university; and acknowledges a Center of Excellence grant from the 10.13039/100008444Parkinson Foundation. 10.13039/501100004267RAH’s university has received research support from Annovis Bio, Inc., Artizan Biosciences, Parkinson’s & Movement Disorder 10.13039/100027925Alliance, Inhibikase Therapeutics, AbbVie, Inc., 10.13039/100005614Biogen MA, Bukwang Pharmaceutical Co., Ltd., Cavion, Inc., Cerevance, Inc., 10.13039/100019850Cerevel Therapeutics, Cynapsus Therapeutics, Enterin, Inc., Genentech, 10.13039/100008086Global Kinetics Corporation, Hoffman‑La Roche Inc., Impax Laboratories, Integrative Research Laboratories Sweden, 10.13039/501100013327Lundbeck, Inc., 10.13039/100000864Michael J. Fox Foundation for Parkinson’s Research, National Parkinson’s Foundation, Neuraly Inc., 10.13039/100014593Neurocrine Biosciences, Neuroderm, Pharma Two B Ltd, 10.13039/100015516Revance Therapeutics, 10.13039/100014605Sage Therapeutics, Sanofi Pharmaceuticals, 10.13039/501100006118Scion NeuroStim, SunPharma, and UCB BioPharma.

## Funding statement

This review and exploratory post hoc analysis pooled from pimavanserin clinical trials were funded by 10.13039/100009427Acadia Pharmaceuticals, Inc. (San Diego, 10.13039/100030481California).

## CRediT authorship contribution statement

**Ana Berrio:** Writing – review & editing, Supervision, Conceptualization. **Lambros Chrones:** Writing – review & editing, Supervision, Conceptualization. **Victor Abler:** Writing – original draft, Supervision, Conceptualization. **Robert A. Hauser:** Writing – review & editing, Conceptualization.

## Declaration of competing interest

The authors declare that they have no known competing financial interests or personal relationships that could have appeared to influence the work reported in this paper.
